# Supplementary material for: Open design of a reproducible videogame controller for MRI and MEG
Source: PLoS One. 2023 Nov 1;18(11):e0290158. doi: 10.1371/journal.pone.0290158 (PMC10619825; doi:10.1371/journal.pone.0290158)
Supplement: S1 Appendix — (PDF) [file pone.0290158.s001.pdf]

Game controller for fMRI

Release 2020-beta

Courtois NeuroMod team

Sep 02, 2022

CONTENTS:

1 Bill of material 3

2 3D printing the parts 5

3 Building the controller body 15

4 PCB assembly instructions 19

5 Programming the Teensy board 21

This document describes how to build the MR compatible videogame controller developed for the Courtois Neuromod project.

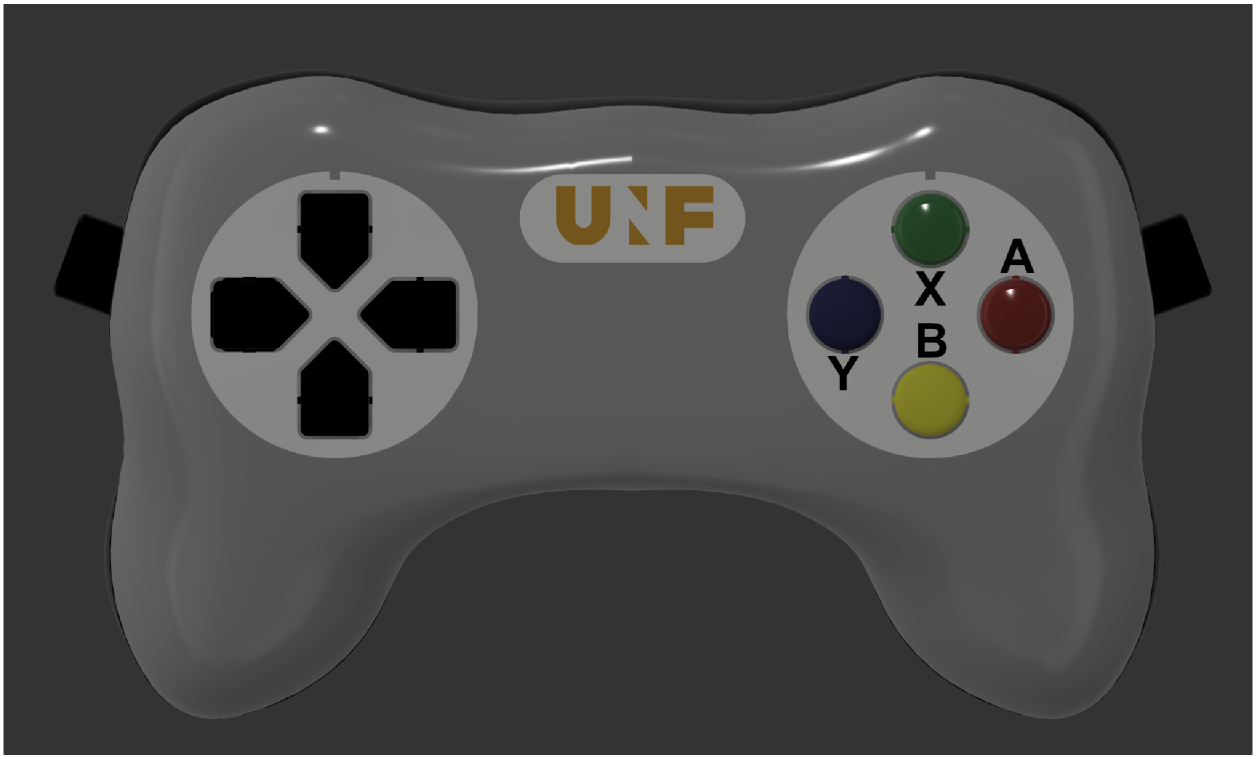

CHAPTER

ONE

BILL OF MATERIAL

This section lists the commercially available parts needed to build the controller. At the time of building, these could be obtained for slightly less than $1000 CAD. When available, the manufacturer's part number is also given to facilitate finding the parts from other suppliers.

Part	Supplier	Manufacturer part no.	Supplier part no.	Qty
**Electronics**				
Fiber optic cable duplex 100m	Digikey	HFBR-RUD100Z	516-2093-ND	1
Fiber optic transmitter	Digikey	SP000063814	516-2213-ND	8
Fiber optic receiver	Digikey	SP000063855	516-2455-ND	8
Teensy 3.5 microcontroller board**It is important to use version 3.5 and not 3.6.I/O pins on 3.6 are not 5V tolerant**	Digikey	DEV-14055	1568-1443-ND	1
Gate driver	Digikey	SN75451BP	296-1746-5-ND	5
Hose 13mm ID, black PE 1	Digikey	CLTS50F-C	298-13576-ND	1
Hose 19.48mm ID, black PE 1	Digikey	CLTS75F-C	298-13577-ND	1
Voltage regulator	Digikey	LM7805CT/NOPB	296-47192-ND	1
Resistor 330	Digikey	ROX1SJ330R	A131459CT-ND	8
Resistor 65	Digikey	SFR16S0006499FR500	PPC64.9XCT-ND	8
Capacitor ceramic 0.1 µF	Digikey	C315C104M5U5TA	399-4151-ND	8
Capacitor ceramic 10 µF	Digikey	C324C106K3R5TA	399-13950-ND	2
Connector header pin breakaway 24 pos	Digikey	4-102972-0-24	A26519-24-ND	4
Connector receptacle 24 pos	Digikey	929850-01-24-RA	929850E-01-24-ND	2
**Hardware**				
Polycarbonate Pan Head Philips Screw 8-32 x 1” 2	McMaster-Carr		93140A782	2
Polycarbonate Pan Head Philips Screw 8-32 x 1/2” 2	McMaster-Carr		93140A588	3
Polycarbonate Pan Head Philips Screw 4-40 x 3/16” 3	McMaster-Carr		93140A403	8
Nylon 6/6 Female Threaded Round Standoff 8-32 x 1/4” 2	McMaster-Carr		96110A026	5
Nylon 6/6 Female Threaded Round Standoff 4-40 x 1/8” 3	McMaster-Carr		96110A001	8

1. Optional
2. Metric equivalent M4 thread
3. Metric equivalent M2.5 thread

CHAPTER

TWO

3D PRINTING THE PARTS

This file details the list of 3D printed parts required to build the controller and gives information about the main printing parameters used. All parts were printed using an Ultimaker 3 printer. The print direction given was set as the positive Z direction in the printer. So if the direction **+Y** is given, the positive Y direction from the .stl file was set as positive Z in the slicing software.

Parts usually require a small amount of post finishing. A kit of fine grit files like this is very useful for that purpose.

2.1 List of files

1. Casing_Top
2. Casing_Bottom
3. Switch_Body
4. Shutter_Cap
5. Shutter_Blade
6. DPad_Support
7. Dir_Button
8. Button_Cap
9. DPad_Cover
10. Button_Cover
11. Interface_Casing_Top
12. Interface_Casing_Bottom

2.2 Casing top

Top.zip	
Weight	53.8 g
Volume	46.4 cm3
Bounding box	155.4 x 43.5 x 94.9 mm
Qty	1
Material 1	PLA
Material 2 (support)	PVA
Material 1 density	100%
Material 2 density	40%
Nozzle 1 size	0.8 mm
Nozzle 2 size	0.4 mm
Nozzle 1 temperature	205 °C
Nozzle 2 temperature	230 °C
Bed temperature	50 °C
Layer height	0.15 mm
Print direction	+Y
Comments	

2.3 Casing bottom

Bottom.zip	
Weight	107.9 g
Volume	87.0 cm3
Bounding box	169.8 x 58.8 x 95.1 mm
Qty	1
Material 1	PLA
Material 2 (support)	PVA
Material 1 density	100%
Material 2 density	40%
Nozzle 1 size	0.8 mm
Nozzle 2 size	0.4 mm
Nozzle 1 temperature	205 °C
Nozzle 2 temperature	230 °C
Bed temperature	50 °C
Layer height	0.15 mm
Print direction	-Y
Comments	

2.4 Switch body

Switch_Body.zip	
Weight	3.6 g
Volume	2.9 cm3
Bounding box	16.0 x 18.5 x 16.0 mm
Qty	8
Material 1	PLA
Material 2 (support)	same as material 1
Material 1 density	100%
Material 2 density	40%
Nozzle 1 size	0.25 mm
Nozzle 2 size	N/A
Nozzle 1 temperature	205 °C
Nozzle 2 temperature	N/A
Bed temperature	50 °C
Layer height	0.1 mm
Print direction	+Y
Comments	

2.5 Shutter cap

Shutter_Cap.zip	
Weight	0.19 g
Volume	0.15 cm3
Bounding box	7.5 x 6.3 x 7.5 mm
Qty	8
Material 1	PLA
Material 2 (support)	-
Material 1 density	100%
Material 2 density	-
Nozzle 1 size	0.25 mm
Nozzle 2 size	-
Nozzle 1 temperature	205 °C
Nozzle 2 temperature	-
Bed temperature	50 °C
Layer height	0.1 mm
Print direction	-Y
Comments	

2.6 Shutter blade

Shutter_Blade.zip	
Weight	0.014 g
Volume	0.0012 cm3
Bounding box	0.5 x 11.7 x 2.2 mm
Qty	8
Material 1	PLA
Material 2 (support)	-
Material 1 density	100%
Material 2 density	-
Nozzle 1 size	0.25 mm
Nozzle 2 size	-
Nozzle 1 temperature	205 °C
Nozzle 2 temperature	-
Bed temperature	50 °C
Layer height	0.06 mm
Print direction	+X
Comments	This works better if printed with dark (ideally black) material. This part takes very little material, prints fast and warps easily, so print lots of it and keep the best ones.

2.7 Direction pad support

DPad_Support.zip	
Weight	14.6 g
Volume	11.8 cm3
Bounding box	46.9 x 13.2 x 48.5 mm
Qty	14
Material 1	PLA
Material 2 (support)	PVA
Material 1 density	100%
Material 2 density	30%
Nozzle 1 size	0.4 mm
Nozzle 2 size	0.4 mm
Nozzle 1 temperature	205 °C
Nozzle 2 temperature	230 °C
Bed temperature	50 °C
Layer height	0.1 mm
Print direction	+Y
Comments	Some post finishing of guiding slots may be required to get smooth button motion. Use of fine grit small detailing files or sanding paper recommended. This part can be printed at lower density than 100%, but make sure that walls are thick enough. Cura's default with 0.4 mm nozzle is 1 mm. At least double that.

2.8 Button support

Buttons_Support.zip	
Weight	16.0 g
Volume	12.9 cm3
Bounding box	46.9 x 13.2 x 48.5 mm
Qty	1
Material 1	PLA
Material 2 (support)	PVA
Material 1 density	100%
Material 2 density	30%
Nozzle 1 size	0.4 mm
Nozzle 2 size	0.4 mm
Nozzle 1 temperature	205 °C
Nozzle 2 temperature	230 °C
Bed temperature	50 °C
Layer height	0.1 mm
Print direction	+Y
Comments	Some post finishing of guiding slots may be required to get smooth button motion. Use of fine grit small detailing files or sanding paper recommended. This part can be printed at lower density than 100%, but make sure that walls are thick enough. Cura's default with 0.4 mm nozzle is 1 mm. At least double that.

2.9 Direction button

Dir_Button.zip	
Weight	1.6 g
Volume	1.3 cm3
Bounding box	14.0 x 12.0 x 13.0 mm
Qty	4
Material 1	PLA
Material 2 (support)	PVA
Material 1 density	100%
Material 2 density	30%
Nozzle 1 size	0.25 mm
Nozzle 2 size	0.4 mm
Nozzle 1 temperature	205 °C
Nozzle 2 temperature	230 °C
Bed temperature	50 °C
Layer height	0.06 mm
Print direction	+Y
Comments	Precision of dimensions and finish quality are very important for this part. Fill percentage does not really matter, but this is a very small part, so material quantity and print time are both are not a problem.

2.10 Round button

Button_Cap.zip	
Weight	1.19 g
Volume	0.96 cm3
Bounding box	10.65 x 13.0 x 13.5 mm
Qty	4
Material 1	PLA
Material 2 (support)	PVA
Material 1 density	100%
Material 2 density	30%
Nozzle 1 size	0.25 mm
Nozzle 2 size	0.4 mm
Nozzle 1 temperature	205 °C
Nozzle 2 temperature	230 °C
Bed temperature	50 °C
Layer height	0.06 mm
Print direction	+Y
Comments	Precision of dimensions and finish quality are very important for this part. Fill percentage does not really matter, but this is a very small part, so material quantity and print time are both are not a problem.

2.11 Direction pad cover

DPad_Cover.zip	
Weight	1.19 g
Volume	0.96 cm3
Bounding box	10.65 x 13.0 x 13.5 mm
Qty	4
Material 1	PLA
Material 2 (support)	PVA
Material 1 density	100%
Material 2 density	30%
Nozzle 1 size	0.25 mm
Nozzle 2 size	0.4 mm
Nozzle 1 temperature	205 °C
Nozzle 2 temperature	230 °C
Bed temperature	50 °C
Layer height	0.06 mm
Print direction	+Y
Comments	

2.12 Button_Cover

Button_Cover.zip	
Weight	1.19 g
Volume	0.96 cm3
Bounding box	10.65 x 13.0 x 13.5 mm
Qty	4
Material 1	PLA
Material 2 (support)	PVA
Material 1 density	100%
Material 2 density	30%
Nozzle 1 size	0.25 mm
Nozzle 2 size	0.4 mm
Nozzle 1 temperature	205 °C
Nozzle 2 temperature	230 °C
Bed temperature	50 °C
Layer height	0.06 mm
Print direction	+Y
Comments	This part uses 2 colors printing for the markings. You need to load 5 stl files in your slicer, assign each to the appropriate material, then merge them into one piece

CHAPTER

THREE

BUILDING THE CONTROLLER BODY

This document describes the steps necessary to build and assemble the hand-held controller body part.

3.1 Assembly

3.1.1 Optical switches

Shutter assembly

Required:

Parts

- Shutter_Blade x 8
- Shutter_Cap x 8
- Switch_Body x 8

Tools

- Cyanoacrylate adhesive
- Fine sanding paper or files
- X-acto knife

Check both shutter blade and shutter cap for smoothness of surface and file or sand any rough surface. Test insertion of blade into slot at the bottom of shutter cap. It should slide to the bottom easily but you should still feel a bit of resistance. Put a drop of cyanoacrylate adhesive on a liquid tight surface, dip the end of shutter blade in adhesive and insert into slot at bottom of shutter cap.

Shutter assembly drawing	Shutter assembly exemple

Switch assembly

1. Disassemble a discarded computer keyboard to recuperate the membrane making the rubber dome switches. Individually cut away the switch dome parts and remove centre of top surface using an x-acto knife.
2. Test assembly of shutter, membrane and switch body by inserting the shutter by inserting the shutter blade into the empty centre part of rubber dome, then into gap at the top of switch body. The shutter blade should completely occult the guide hole for the fiber optic when button is pressed down, but leave it free when in upward position. The distance between the bottom of the shutter cap and the top of switch body will determine the stroke length of the key. The smaller this distance, the more responsive the controls will be. The shutter should move freely up and down.

Shutter up	Shutter pressed down

3. Since the rubber domes recuperated from computer keyboards will very likely vary in dimensions, it might be necessary to modify shutter cap and shutter blade dimensions. The file Shutter_Assembly_Param.f3d contains these two parts in Autodesk Fusion 360 format. The design is parametric to facilitate modifications. To edit the parts, open the file in **Fusion 360**, go to Modify, then select change parameters and expand User Parameters. Changing the value of parameter *Shutter_Cap_Shaft_Length* will allow to adjust the stroke length, while changing *Shutter_Blade_L* will allow ensuring correct shutter operation. That part is also available in .step format for import in other CAD programs.

4. Ensure that the air gap and the fiber optic guide in the 3D printed switch bodies are free of any residual PLA or support material. Hold switch body in front of a light source and check along Z axis for the air gap, and along horizontal axis for the fiber optic guide.

3.1.2 Controller body

1. For each control on the controller, cut one length of duplex fiber optic to desired length between controller and interface module + 150 mm
2. Split each length of fiber on its length to separate the 2 fibers on a distance of approx. 750 mm from one end, and 25 mm from second end
3. Label each fiber with the name of matching control. On the end where the fibers are split for the largest distance (controller end) place a label on each single fiber
4. On the controller side of the fibers, make two bundles of fibers. Place one fiber of eac78h control in either bundle.
5. For one of the two bundles, cut the fibers for the directional pad controls about 150 mm shorter than the ones for the buttons. Do the opposite with the other bundle. This will allow a cleaner layout of the fibers inside the casing.
6. Strip and polish fibers
7. Cut two 500 mm lengths of the 13 mm ID plastic hose and fix those on the ends of the Y splitter
8. Fix second end of plastic hoses to extrusions on part *Casing_Bottom*

Fiber_Sheath_Assembly

9. For each control, place a *Switch_Body* in matching groove inside casing.
10. Insert the fiber optics into Y splitter and push gently until both fibers emerge inside of casing. The bundle where the buttons fibers are longer will go on the directional pad side. There will be one fiber for each control in each

branch of the Y. Make sure that the length protruding inside the casing is enough to reach into the corresponding *Switch_Module* and allow to lay out cleanly inside casing.

11. For each control, glue both fibers in place **(Need glue specs)**
12. Optional: Depending on fit tightness of *Switch_Module* parts inside grooves, it is also possible to glue the module in place. If gluing modules in place, wait for glue on fibers to set before proceeding
13. Place one threaded Nylon insert in each mounting post of part *Casing_Top*. If inserts don't fit inside, re-drill hole using 1/4" (6.35 mm) bit. If inserts are loose, use a drop of cyanoacrylate adhesive

14. Before starting assembly, test bottom and top parts of casing for fit. The two halves should fit together without any visible gap. Common area that might need post processing are the top and bottom of screw posts and the lip that runs around the perimeter of the bottom part, as well as the matching flat surface on the top part. If necessary, file or sand away excess of material that might prevent case from closing properly.

Test casing for fit

| Areas commonly requiring post-processing

3.1.3 Optical fibers assembly

Parts ready for assembly

1. Separate bundle of fiber optics into two smaller bundles of 8 fibers each. Pull each smaller bundle through the Y splitter with one small bundle coming out of each of the Y branches. Pull each of the smaller bundle into the 1 meter protective sheath until there is approximately 300 mm of fibers coming out of each sheath.

Separating fibers bundle with Y splitter

2. Pull each bundle into the bottom part of casing through openings on the sides and make sure that the sheaths fit nicely on the protruding parts and that you have at least 200 mm length of fibers going into the casing on both sides.

Fibers insertion in casing

3. Strip fibers and polish the fibers. It's possible to use a regular wire stripping tool set for #18 wire size. Insert fibers into switch body so teh tip of the fiber is flush with the internal wall of the air gap. Ensure that the shutter is free to move after the fibers are in place.
4. Use a drop a glue to affix the fibers to the switch body. The type of glue is not critical, but **do not use** cyanoacrylate or other type of *permanent* glue. These will work, but they will make the switch impossible to repair, which means in case of a broken fiber or other problem, you will need to replace the whole switch.

Fibers connections and testing

5. To assemble the switch inside the casing, it is easier to proceed by pairs, waiting for the glue on the fibers to set before proceeding to the next pair. Proceeding that way allows to move the fibers around to route them cleanly inside the casing without pulling them out from the body of already installed switches. The order is not critical, but it is a lot easier to finish with the pair that's the closest to the opening where the fibers enter the casing.

Assembling first pair of controls

Assembling second pair of controls

Assembling third pair of controls

Assembling fourth pair of controls

Add buttons supports

Installing rubber membranes and shutters

Finished view

Re-install buttons supports

Close casing

Casing closed top view

Install buttons

Finished view

Install direction pad cover and buttons cover

Assembly complete

CHAPTER

FOUR

PCB ASSEMBLY INSTRUCTIONS

Get PCB 3D model here

4.1 Ordering PCBs

Get Gerber files here and send to fab house

4.2 Assembly

Raw PCB view

PCB isometric view

PCB Top view

PCB Side view

PCB Side view

Teensy with headers	Teensy on PCB

The Teensy board can be mounted either on headers (left) or directly on PCB (right). The first option is recommended as is allows easy replacement in case of damage.

Adjust potentiometer to be 330 between the two illustrated pins or solder directly a 330 resistor between those pins.

Board layout

Schematics	Component	Value
IC1 - IC5, IC7	Gate driver	SN75451BP
IC6	Voltage regulator	LM340
R1 - R10	Resistor	65
R11 - R29	Resistor	330 between pin 1 & 3
R21	Resistor	5 k
C1 - C8	Capacitor	0.1 µF
C11,C12	Capacitor	10 µF
TX1 - TX10	Fiber optic transmitter	SFH756V
RX1 - RX10	Fiber optic receiver	SFH551V
Teensy 3.5	Use version 3.5 **NOT 3.6**	

TX9,TX10,RX9,RX10 and associated components are optional. They were meant for additional controls not implemented on this version of the controller. They can also be used for troubleshooting purpose or to fix a damaged channel.

CHAPTER

FIVE

PROGRAMMING THE TEENSY BOARD

The code running on the microcontroller was written with the Arduino IDE using the teensyduino add-on.

5.1 Arduino

The Arduino IDE can be downloaded here.

5.2 Teensyduino

Download and install the Teensyduino add-on

5.3 Code

Upload the code on the Teensy board by cutting and pasting the code block below in Arduino IDE or downloading the file Neuromod_Game_Controller.ino and opening it. Then select the board Teensy 3.5 and USB type Keyboard in the Tools menu. After this is done, click the upload button.

```
/*
    Name:       Neuromod_Game_Controller.ino
    Created:        2019-01-08 11:22:35
    Author:     Andre Cyr
*/

#include <usb_keyboard.h>

char keyVal[] = {'A','B','X','Y','U','R','D','L'};
char trgKeyVal = '5';
uint8_t rxPin[] = { 24,25,26,27,28,29,30,31 };
uint8_t nPin = sizeof(rxPin);
uint16_t rxPinState[sizeof(rxPin)];
uint8_t trgPin = 2;
uint8_t txPin[] = {14,15,16,17,18,19,20,21,22,23};
uint16_t timeKeyPressed;
bool buttonPressed[sizeof(rxPin)];
```

(continues on next page)

(continued from previous page)

```
bool buttonToggled[sizeof(rxPin)];
bool trigKeyPressed;
volatile bool triggered = false;
bool debug;
uint8_t ledPin = 13;
uint8_t trgKeyPressLength;

// The setup() function runs once each time the micro-controller starts
void setup()
{
        pinMode(trgPin, INPUT);
        pinMode(ledPin, OUTPUT);
        debug = digitalRead(trgPin) == 0 ? true : false;
        trgKeyPressLength = 10; // duration of key press for trigger in ms

        for (int i = 0;i < nPin;i++)
        {
                pinMode(rxPin[i], INPUT);
                rxPinState[i] = 0;
                buttonPressed[i] = true;
                buttonToggled[i] = true;
        }

        for (int i = 0; i < nPin; i++)
        {
                pinMode(txPin[i], OUTPUT);
                digitalWrite(txPin[i], HIGH);
        }

        attachInterrupt(trgPin, trgIn, RISING);

        delay(1000); //required for PC to properly detect keyboard

        Keyboard.println("Initializing...");
        Keyboard.print("Trigger key = ");
        Keyboard.println(trgKeyVal);
        for (int i = 0;i < nPin;i++)
        {
                bool pinDetected = digitalRead(rxPin[i]) ? false : true;
                if (pinDetected)
                {
                        Keyboard.print("Key ");
                        Keyboard.print(keyVal[i]);
                        Keyboard.println(" : OK ");
                }
                else
                {
                        Keyboard.print("Key ");
                        Keyboard.print(keyVal[i]);
                        Keyboard.println(" : not detected ");
                }
        }
```

(continues on next page)

(continued from previous page)

```
}

// Add the main program code into the continuous loop() function
void loop()
{

        if (debug)
        {
                // write debug code here
                // debug mode is activated by pulling pin 2 (trigger pin) low at start-up


        }

        else
        {
                if (triggered)
                {
                        Keyboard.press(trgKeyVal);
                        timeKeyPressed = millis();
                        trigKeyPressed = true;
                        triggered = false;
                }
                else
                {
                        if (trigKeyPressed)
                        {
                                if ((millis() - timeKeyPressed) > 10)
                                {
                                        Keyboard.release(trgKeyVal);
                                        trigKeyPressed = false;
                                }
                        }
                }


                // Sequentially reads each key state and writes state as LSB in␣
↪rxPinState[i]
                // shiftings bits left by 1 bit

                for (int i = 0;i < nPin;i++)
                {
                        rxPinState[i] = (rxPinState[i] << 1) | (1 &␣
↪digitalRead(rxPin[i]));
                }

                for (int i = 0;i < nPin;i++)
                {

                        // The key state will be considered pressed and debounced if all␣
↪bits are 1s,
                        // or released and debounced if all bits are 0s (16 identical␣
```

(continues on next page)

(continued from previous page)

```
↪readings)

                        if (rxPinState[i] & 65535)
                        {
                                buttonToggled[i] = buttonPressed[i] ? false : true;
                                buttonPressed[i] = true;

                        }
                        if (!rxPinState[i])
                        {
                                buttonToggled[i] = buttonPressed[i] ? true : false;
                                buttonPressed[i] = false;
                        }
                }

                for (int i = 0;i < nPin;i++)
                {
                        if (buttonPressed[i] & buttonToggled[i])
                        {
                                Keyboard.press(keyVal[i]);
                                buttonToggled[i] = false;
                        }
                        if (!buttonPressed[i] & buttonToggled[i])
                        {
                                Keyboard.release(keyVal[i]);
                                buttonToggled[i] = false;
                        }
                }
        }
}

void trgIn()
{
        triggered = true;
}
```
