# Supplementary figures and images for: Open design of a reproducible videogame controller for MRI and MEG
Source: PLoS One. 2023 Nov 1;18(11):e0290158. doi: 10.1371/journal.pone.0290158 (PMC10619825; doi:10.1371/journal.pone.0290158)

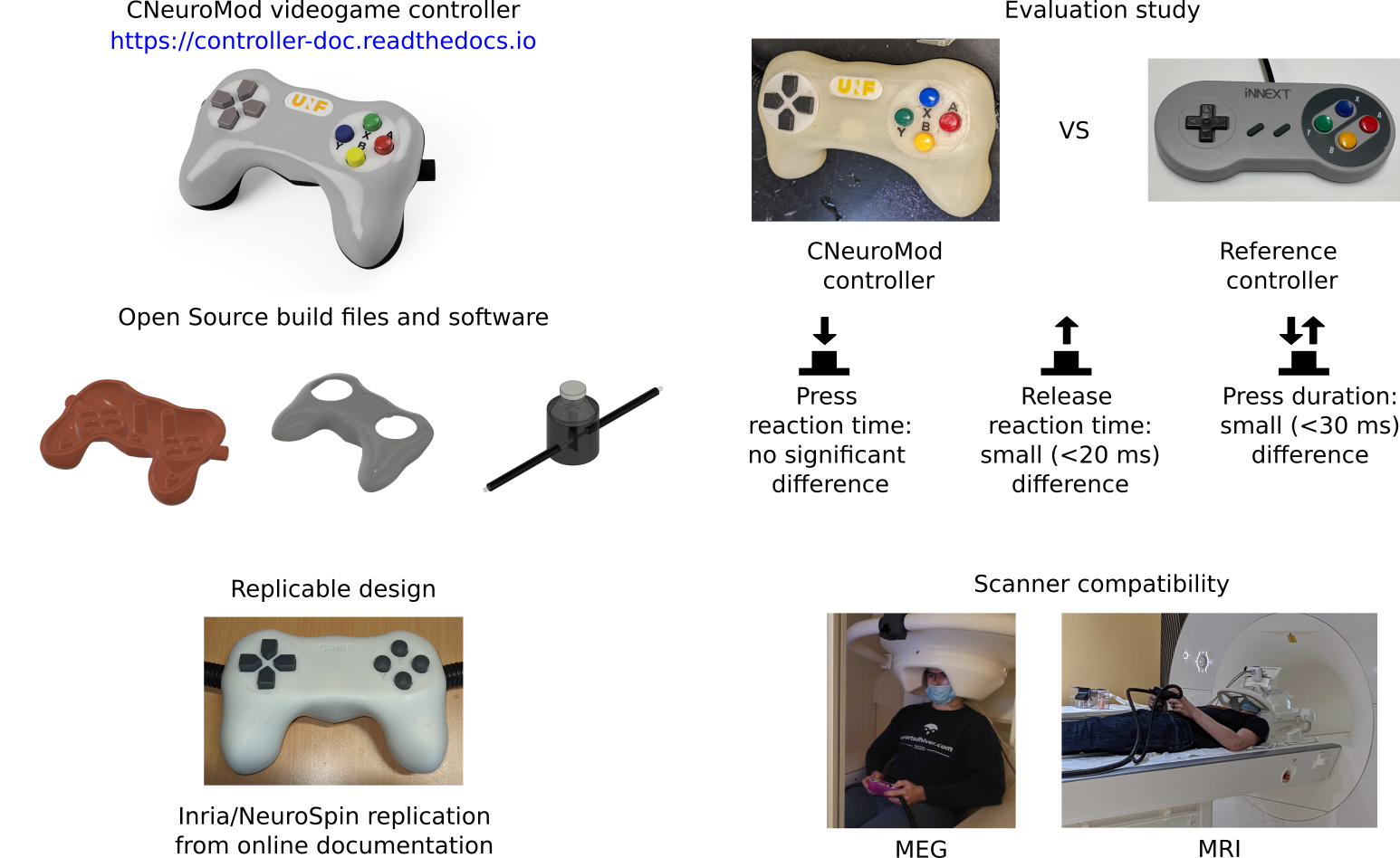

Supplement: S1 Graphical abstract — (TIF) [file pone.0290158.s008.tif]
