# Supplementary material for: Open design of a reproducible videogame controller for MRI and MEG
Source: PLoS One. 2023 Nov 1;18(11):e0290158. doi: 10.1371/journal.pone.0290158 (PMC10619825; doi:10.1371/journal.pone.0290158)
Supplement: Attachment — Submitted filename: response_letter.docx [file pone.0290158.s009.docx]

# Response to reviewers

We thank the editor for providing us the opportunity to revise our work, and the reviewers for the insightful comments which have helped improve our study. A point-by-point response to reviewers can be found below.

# Formatting :

1. Please ensure that your manuscript meets PLOS ONE's style requirements, including those for file naming. The PLOS ONE style templates can be found at
<https://journals.plos.org/plosone/s/file?id=wjVg/PLOSOne_formatting_sample_main_body.pdf> and
<https://journals.plos.org/plosone/s/file?id=ba62/PLOSOne_formatting_sample_title_authors_affiliations.pdf>

Authors answer : We reformatted the manuscript to follow the PLOS ONE.

2. We note that the grant information you provided in the ‘Funding Information’ and ‘Financial Disclosure’ sections do not match.

When you resubmit, please ensure that you provide the correct grant numbers for the awards you received for your study in the ‘Funding Information’ section.

Authors answer : The information provided in the Financial Disclosure section of the submission has been re-verified and is correct.

3. Thank you for stating the following in the Acknowledgments Section of your manuscript:
"The Courtois project on neural modeling was made possible by a generous donation from the Courtois foundation, administered by the Fondation Institut Gériatrie Montréal at CIUSSS du Centre-Sud-de-l’île-de-Montréal and University of Montreal. The Courtois NeuroMod team is based at “Centre de Recherche de l’Institut Universitaire de Gériatrie de Montréal”, with several other institutions involved. See the CNeuromod documentation for an up-to-date list of contributors ([https://docs.cneuromod.ca](https://docs.cneuromod.ca/)). The replication of the controller by the MIND team, Inria, France has received funding from the European Union’s Horizon 2020 Framework Programme for Research and Innovation under the Specific Grant Agreement No. 945539 (Human Brain Project SGA3) and through the joint Inria “NeuroMind” team grant to PB, KJ, BT and Alexandre Gramfort. PB is a senior fellow (“chercheur boursier senior”) of the “Fonds de recherche du Québec - Santé”. KJ is supported by funding from the Canada Research Chairs program and a Discovery Grant from the Natural Sciences and Engineering Research Council of Canada.
The authors would like to thank the MEG Imaging Center and Courtois-Neuromod scanning crews for acquiring the validation data. "

We note that you have provided funding information that is not currently declared in your Funding Statement. However, funding information should not appear in the Acknowledgments section or other areas of your manuscript. We will only publish funding information present in the Funding Statement section of the online submission form.
Please remove any funding-related text from the manuscript and let us know how you would like to update your Funding Statement. Currently, your Funding Statement reads as follows:
"The Courtois project on neural modeling was made possible by a generous donation from the Courtois foundation, administered by the Fondation Institut Gériatrie Montréal at CIUSSS du Centre-Sud-de-l’île-de-Montréal and University of Montreal. The Courtois NeuroMod team is based at “Centre de Recherche de l’Institut Universitaire de Gériatrie de Montréal”, with several other institutions involved. See the CNeuromod documentation for an up-to-date list of contributors ([https://docs.cneuromod.ca](https://docs.cneuromod.ca/)). The replication of the controller by the MIND team, Inria, France has received funding from the European Union’s Horizon 2020 Framework Programme for Research and Innovation (<https://research-and-innovation.ec.europa.eu/funding/funding-opportunities/funding-programmes-and-open-calls/horizon-2020_en>) under the Specific Grant Agreement No. 945539 (Human Brain Project SGA3) and through the joint Inria “NeuroMind” team grant to PB, KJ, BT and Alexandre Gramfort. PB is a senior fellow (“chercheur boursier senior”) of the “Fonds de recherche du Québec - Santé”. KJ is supported by funding from the Canada Research Chairs (<https://www.chairs-chaires.gc.ca/home-accueil-eng.aspx>) program and a Discovery Grant from the Natural Sciences and Engineering Research Council of Canada (<https://www.nserc-crsng.gc.ca/index_eng.asp>). The funders had no role in study design, data collection and analysis, decision to publish, or preparation of the manuscript."

Please include your amended statements within your cover letter; we will change the online submission form on your behalf.

Authors answer : We removed the paragraph related to funding information in the “Acknowledgement” section. The information (“Financial Disclosure”) provided in the submission form were verified and are correct.

4. In your Data Availability statement, you have not specified where the minimal data set underlying the results described in your manuscript can be found. PLOS defines a study's minimal data set as the underlying data used to reach the conclusions drawn in the manuscript and any additional data required to replicate the reported study findings in their entirety. All PLOS journals require that the minimal data set be made fully available. For more information about our data policy, please see <http://journals.plos.org/plosone/s/data-availability>.

"Upon re-submitting your revised manuscript, please upload your study’s minimal underlying data set as either Supporting Information files or to a stable, public repository and include the relevant URLs, DOIs, or accession numbers within your revised cover letter. For a list of acceptable repositories, please see <http://journals.plos.org/plosone/s/data-availability#loc-recommended-repositories>. Any potentially identifying patient information must be fully anonymized.

Important: If there are ethical or legal restrictions to sharing your data publicly, please explain these restrictions in detail. Please see our guidelines for more information on what we consider unacceptable restrictions to publicly sharing data: <http://journals.plos.org/plosone/s/data-availability#loc-unacceptable-data-access-restrictions>. Note that it is not acceptable for the authors to be the sole named individuals responsible for ensuring data access.

We will update your Data Availability statement to reflect the information you provide in your cover letter.

Authors answer : We saved the cleaned versions of the datasets used to generate the figures as .csv files (and a .pkl file for MEG covariance matrices) and added them to Supporting Information.

Due to restrictions from the ethics committee in charge of the study, human neuroimaging data is only accessible through a registered access model in a databank hosted at our institution. The instructions to apply for access to the data can be found here: https://www.cneuromod.ca/access/access/

5. Please include your full ethics statement in the ‘Methods’ section of your manuscript file. In your statement, please include the full name of the IRB or ethics committee who approved or waived your study, as well as whether or not you obtained informed written or verbal consent. If consent was waived for your study, please include this information in your statement as well.

Authors answer : We modified the manuscript to add a section named “2.2.2 Ethics statement”, containing the information provided in the submission form.

6. We note that Figures 1,2 and 3in your submission contain copyrighted images. All PLOS content is published under the Creative Commons Attribution License (CC BY 4.0), which means that the manuscript, images, and Supporting Information files will be freely available online, and any third party is permitted to access, download, copy, distribute, and use these materials in any way, even commercially, with proper attribution. For more information, see our copyright guidelines: <http://journals.plos.org/plosone/s/licenses-and-copyright>.

We require you to either (1) present written permission from the copyright holder to publish these figures specifically under the CC BY 4.0 license, or (2) remove the figures from your submission:

a. You may seek permission from the original copyright holder of Figures 1, 2, and 3 to publish the content specifically under the CC BY 4.0 license.

We recommend that you contact the original copyright holder with the Content Permission Form (<http://journals.plos.org/plosone/s/file?id=7c09/content-permission-form.pdf>) and the following text:
“I request permission for the open-access journal PLOS ONE to publish XXX under the Creative Commons Attribution License (CCAL) CC BY 4.0 (<http://creativecommons.org/licenses/by/4.0/>). Please be aware that this license allows unrestricted use and distribution, even commercially, by third parties. Please reply and provide explicit written permission to publish XXX under a CC BY license and complete the attached form.”

Please upload the completed Content Permission Form or other proof of granted permissions as an "Other" file with your submission.

In the figure caption of the copyrighted figure, please include the following text: “Reprinted from [ref] under a CC BY license, with permission from [name of publisher], original copyright [original copyright year].”

b. If you are unable to obtain permission from the original copyright holder to publish these figures under the CC BY 4.0 license or if the copyright holder’s requirements are incompatible with the CC BY 4.0 license, please either i) remove the figure or ii) supply a replacement figure that complies with the CC BY 4.0 license. Please check copyright information on all replacement figures and update the figure caption with source information. If applicable, please specify in the figure caption text when a figure is similar but not identical to the original image and is therefore for illustrative purposes only.

Authors answer : The figure 1 contains only images we created ourselves and for which we own the full rights (both pictures and 3d models). The figures 2 and 3 have been re-created from scratch using only original images for which we own the full rights.

7. Please include captions for your Supporting Information files at the end of your manuscript, and update any in-text citations to match accordingly. Please see our Supporting Information guidelines for more information: [http://journals.plos.imagesorg/plosone/s/supporting-information](http://journals.plos.org/plosone/s/supporting-information).

Authors answer : We added a section named Supporting Information that mentions the documentation and dataset files.

# Content :

1a. The motivation to build a scanner-compatible videogame controller in-house and publish a thorough engineering analysis of the project is apparent in the Introduction, but it can be stated in more complete fashion.

Based on the prototype, the in-house device costs approximately CAD$1,000 in parts and an undefined amount of experienced labor to build. A bit of investigation reveals that the industry standard Current Designs scanner-compatible videogame controller costs approximately US$4,600, plus fees. The authors appear to justify their project by offering a less costly solution, but a detailed calculation is not presented to support this justification.

Authors answer : We thank the reviewer for pointing out the need for a more thorough price comparison. We modified the paragraph named “Open design and reusability” to add a table (Table 5) that breaks down the prices of the bill of materials. The Discussion has been modified to also discuss the price of a commercial vs open solution.

1b. A further concern about the relative cost as a justification to build a controller in-house is that the comparability of the haptics and user satisfaction between the device alternatives is not addressed. The participant sample in the results is small and from a narrow age range. Although a larger cohort from a much broader age range would be desirable, some sort of utility rating (as collected from the MIND Team confirmation study) is really needed to fully support the conclusion that the CNeuroMod controller is a suitable substitute for the commercially proven and available alternative.

Authors answer : The utility ratings of the CNeuroMod sample (i.e. the subjects who performed the cued-response task) were added to the paper as well as the answers of the Inria/Neurospin participants. These additional data are now presented in the *Utility ratings* sections of the Methods and Results. The Discussion has been modified to relate utility ratings to other behavioral results, and the ratings dataset has been added to Supporting Information.

2. Release reaction times on long trials were significantly longer with the prototype than the mock condition commercial controller (non-scanner compatible). No explanation is offered in the Results or Discussion.

Why is there an apparent difference in the responsiveness of the prototype, depending upon duration of the button presses? How does this responsiveness relate to the poor rating from the MIND Team confirmation study participants (i.e., “a value of 2 for the following items: smoothness during operation and general comfort.”)?

Authors answer : We discuss this effect under the section named “Reactivity of the CNeuroMod videogame controller”. We agree that the wording did not make clear that we were addressing specifically the longer release times of our prototype, so we modified the text to make it more explicit. We also added a sentence stating that this effect would likely contribute to the poor utility ratings.

3. The title and text refer to a validation of the original test results, which had showed the comparability of the functional values from the prototype with a non-scanner compatible mock-up device. No true validation study is reported. In other words, there are no results from two or more cohorts performing the same tests as sequential experiments, and there is no analysis of validating results (i.e., an independent samples t-test, for example).

The MIND Team “study” is relevant, but not well described. The measures in the main results do not appear in the MIND Team section (3.4), and the Mind Team user ratings do not appear in the main results. This is not a replication, and referring to the MIND Team information as a replication needs to corrected throughout.

Authors answer : We agree with the reviewer that the terms “replication” and “validation” were used improperly. We removed the term validation from the title. We also replaced references to this study as “validation” for other terms, such as “feasibility study”. We replaced references to “replication” by “reproduction” of the controller device, because the study wasn’t replicated by the MIND team, but they produced a second controller unit.

4. The manuscript reads clearly enough, although the voice or writing style changes noticeably between sections of the text. Conforming the entire report to one writing style would improve its readability.

Authors answer : The style and voice of the manuscript have been reviewed and corrected by one of the authors who is a native English speaker.

5. Figure 8 includes panels A through L. The legend for Figure 8 only explains panels A through J.

Panels K and L should either be explained in the legend and text, or eliminated from the figure.

Authors answer : The legend was corrected to cover panels A through L.

6. Overall the paper was quite straightforward and clearly addresses the core question the team started out with. I have one methodological question with regard to how the number of participants and their demographics was chosen. 4 participants isn't very many obviously and the age range of the participants isn't necessarily a good match for what I'd expect of most future work on gaming/neuroimaging. So it'd be useful for the authors to speak to how they determined the sample size and what types of criteria for participants were considered. I'd note though that because I would call this a "feasibility" illustration, the small sample size & potential non-representativeness of the participants aren't "fatal flaws"; but it would be valuable for readers to know how these factors were chosen.

Authors answer : We modified the “Participants” section to explain that the subjects were recruited as part of a larger, longitudinal dataset acquisition for which the controller has been developed. We also included a sentence to state that we expected the task to reflect the controllers capability irrespective of videogame experience.

7. The other issue that I think would improve the paper is a deeper dive into what I would call the exceptionally dated configuration of the controller. The authors indicate that, "Key features of the SNESTM controller can still be found in modern systems such as the XBoxTM (Microsoft) and PlayStationTM (Sony)" which is true, but still reasonably misleading. For instance, it's a bit like if the team created a new car that was basically a Model T Ford, but said that it "still contains many features of modern cars such as the 2023 Honda Accord." It might be true (e.g., it would have 4 wheels, a steering wheel, a windshield), it would still be super dated. Video game controllers have used a dual stick setup since the late 1990s. There are essentially no modern games that I can think of that someone would be able to play with the controller developed here - certainly none of the games used in the research that the authors reference in the introduction (i.e., most of that work on video games/neuroscience has been after the year 2000). So the authors should spend some time on that particular limitation (or else on why it isn't a limitation).

Authors answer : We agree that our description of the controllers main features could be misleading. To clarify, we adjusted paragraph 2.1.1. to remove the reference to modern titles and state that the goal behind the design of this controller was to enable retro videogame play, and not necessarily modern titles. We additionally modified the discussion section called “Implications and future work” to explicitly state that future improvements of the design are going to be required to play modern titles.
